# Application of Patient‐Specific PCL/*β*TCP Scaffolds for Reconstruction of Maxillomandibular Bone Defects: A Case Series

**DOI:** 10.1155/crid/6392381

**Published:** 2026-05-18

**Authors:** Hekmat Farajpour, Helia Sadat Haeri Boroojeni, Sadra Mohaghegh, Hanieh Nokhbatolfoghahaei, Arash Khojasteh

**Affiliations:** ^1^ Deparment of Artificial Intelligence, Smart University of Medical Sciences, Tehran, Iran, smums.ac.ir; ^2^ Artificial Intelligence in Medical Sciences Research Center, Smart University of Medical Sciences, Tehran, Iran, smums.ac.ir; ^3^ Dental Research Center, Research Institute of Dental Sciences, Shahid Beheshti University of Medical Sciences, Tehran, Iran, sbmu.ac.ir

**Keywords:** bone regeneration, computer-aided design, computer-aided manufacture, polycaprolactone, scaffolds, tricalcium phosphate

## Abstract

**Aim:**

This case series study was aimed at reporting reconstruction of maxillomandibular large bone defects using patient‐specific polycaprolactone/*β*‐tricalcium phosphate (PCL/*β*TCP) scaffolds.

**Material and Method:**

In three patients who underwent mandibular segmental resection, scaffolds were implanted to augment load‐bearing areas while preserving the inferior alveolar neurovascular bundle continuity (functional bone replacement). In two other patients, PCL/TCP scaffolds were implanted in non‐load‐bearing areas to separate anatomic spaces (anatomic bone replacement). One anatomic scaffold was used as a covering membrane in posterior mandible atrophic ridge augmentation, and another was used to separate the orbital cavity from the antrum. Each patient underwent computed tomography (CT) scans before surgery and at the end of the follow‐up period. Histological analyses were performed on biopsy samples collected from those who underwent placement of dental implants.

**Results:**

Radiologic analyses showed 30%–75% new bone formation in functional bone replacement. A proper bony border was created following anatomic bone replacement. Minor graft exposure and inflammation were observed in three patients. Histological analyses revealed proper scaffold degradation and bone formation.

**Conclusion:**

Given the overall clinical complications in our study, PCL/*β*TCP patient‐specific scaffolds can be potential treatment options in selected cases. However, further studies are warranted.

## 1. Introduction

Given the imposed suffering that grafting methods can potentially cause, several alternative approaches, such as regenerative methods, have been proposed. Successful bone regeneration is associated with defect size, location, and patients′ age and health status [1]. Among different regenerative approaches, the successful application of tissue scaffolds has been shown previously. The current literature mostly describes scaffold application in localized bone defects of the maxillofacial region (i.e., < 6 cm) [2, 3].

Development of new medical image acquisition methods and multiscale computational and technological advances has contributed to the advent of supplementary and/or alternative patient‐specific scaffolds [3–5]. Patient‐specific scaffolds for bone regeneration are rooted in the implantation of additively manufactured biodegradable scaffolds that induce bone regeneration and preserve a protected healing space that encourages physiologic bone formation and remodeling [6]. Scaffolds that degrade in tune with the host body′s rate of bone formation can provide a temporary environment for facilitated bone regeneration [7–9]. They can be designed according to the initial defect topography and patient requirements using computer‐aided design/computer‐assisted manufacturing (CAD/CAM) technology.

A blend of polycaprolactone (PCL)/beta‐tricalcium phosphate (*β*TCP) provides apt physicochemical and biological properties as well as a chemical composition that resembles native bone tissue [10–15]. Several in vitro and in vivo studies approved the proper osteoinducing ability of the printed PCL/*β*TCP scaffolds [16–19]. However, scarce studies concerning its clinical application are present [3, 20]. These scaffolds are more commonly fabricated through fused deposition modeling (FDM), which has acceptable clinical accuracy and accessibility [21].

In this study, we present a series of five cases undergoing implantation of FDM‐fabricated PCL/*β*TCP patient‐specific scaffolds in both load‐bearing and non‐load‐bearing bone defects of the maxillomandibular region. Three‐dimensional computed tomography (CT) scans were obtained for quantitative evaluation of bone formation and scaffold degradation, and the occurrence of postoperative complications was evaluated clinically during the follow‐up period. Biopsy samples were collected and histologically analyzed upon re‐entry for implant placement. Our results showed that these scaffolds can be a possible treatment option; however, studies with higher sample sizes are required.

## 2. Material and Method

This was a retrospective case series study on patients treated with predesigned 3D‐printed scaffolds. The study was performed according to the World Medical Association Declaration of Helsinki′s declared ethical principle under the approval of the Ethical Committee of the Shahid Beheshti University of Medical Sciences (IR.SBMU.RETECH.REC.1398.377). All patients were informed about surgical procedures, treatment options, and potential complications prior to signing informed consent.

### 2.1. Selection Criteria and Intervention

**Figure 1:**
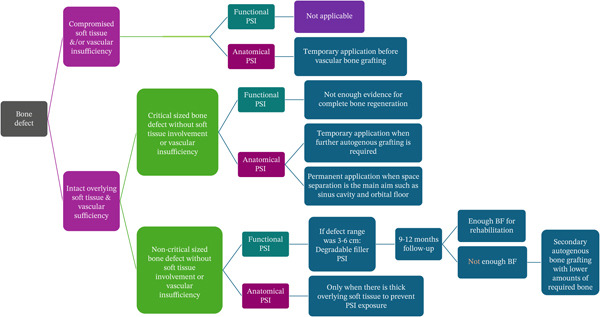
Decision tree to decide whether to perform a regenerative procedure in patients or not.

Patients who underwent segmental or partial resection in the maxillomandibular region were included. Patient selection and treatment planning were performed based on a decision tree shown in Figure 1. Included patients had American Society of Anesthesiologists (ASA) I physical status and were not pregnant or breastfeeding. Patients who could undergo immediate autogenous bone grafting after resection and those who refused to receive regenerative scaffolds were excluded.

PCL/*β*TCP patient‐specific scaffolds were implanted in all defects. The performed surgical procedures entailed two approaches (Table 1): (A) implantation of PCL/*β*TCP scaffolds to induce bone regeneration and augmentation while engulfing the neurovascular bundle (i.e., functional bone replacement for Patients 1, 2, and 3) and (B) applying scaffolds to separate anatomical spaces, such as the antrum, and/or preserve a protected healing space (i.e., anatomical bone replacement for Patients 4 and 5). Each patient underwent CT scans before surgery and at the end of the follow‐up period.

**Table 1:** Characteristics of the patients included in the study.

Patient number	Sex	Age (year)	Type of disease	Surgical methods	Type of scaffold	Defect location	Follow‐up (months)	Missed bone volume	Formed bone volume	Bone formation (%)
1	F	18	Tumor (cemento‐ossifying fibroma)	Bundle preserved	PCL/*β*TCP	Right mandibular body (3 cm length)	12	6219	3421	55.00
2	M	24	Tumor (ameloblastoma)	Bundle preserved	PCL/*β*TCP	Left mandibular ramus and body (6 cm)	15	7108	2133	30.00
3	M	31	Cyst (OKC)	Bundle preserved	PCL/*β*TCP	Right mandibular ramus and body (6 cm)	16	5063	3796	75.00
4	M	22	Cyst (OKC)	NA	PCL/*β*TCP	Left hemimaxilla (7 cm)	18	4503	1577	Successful space separation
5	F	35	Ridge atrophy	NA	PCL/*β*TCP	Left postmandible (3 cm). The scaffold is used as a membrane to cover the defect	16	706	71	Successful border formation

### 2.2. Fabrication of PCL/*β*TCP Scaffolds

**Figure 2:**
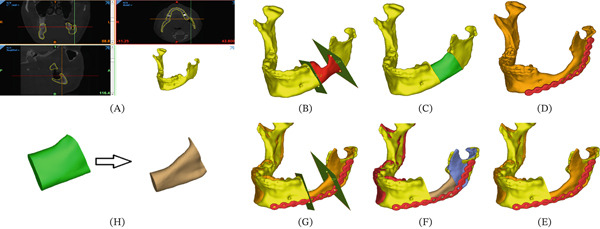
Workflow of measuring the volume of new bone formation. (A) Primary model of the patient. (B) Design of the osteotomy cuts. (C) Designed scaffold to fill the defect. (D) The postsurgical model of the mandible after the follow‐up. (E, F) Superimposing the pre‐ and postoperative models. (G) Recreating the cuts to create an identical region of interest. (H) Comparison of the dissected section. The green one is related to the scaffolds, which are equal to the defect volume, and the brown one is related to the de novo bone. Adapted from [22]. Used with permission from Springer Nature.

FDM technology was used to fabricate scaffolds. The contour of the contralateral site was imitated for scaffold design (Figure 2). For printing, the PCL granules were heated at 125°C, and *β*TCP powder was added till a 60% *w*/*w* *β*TCP blend was obtained. The blend was stirred for 1 h at 30°C. Then, the blend was printed under the following conditions: nozzle speed 1.6 mm/s, pressure 8 bars, and nozzle temperature 160°C. Layer thickness was set at 400 *μ*m, and porosity was set at 50%. Scaffolds were sterilized via gamma irradiation. The proper physicochemical and biological behavior of scaffolds was described in our previous study [19].

### 2.3. Surgical Procedure

#### 2.3.1. Resection

Traditional surgical protocols indicate a 1‐year gap between the resection and reconstructive surgeries. However, our protocol entailed immediate scaffold implantation after resection. Affected tissues were resected using customized surgical guides fabricated based on FDM‐fabricated patient models. All surgical procedures were performed under general anesthesia with nasal intubation.

#### 2.3.2. Functional Bone Replacement

**Figure 3:**
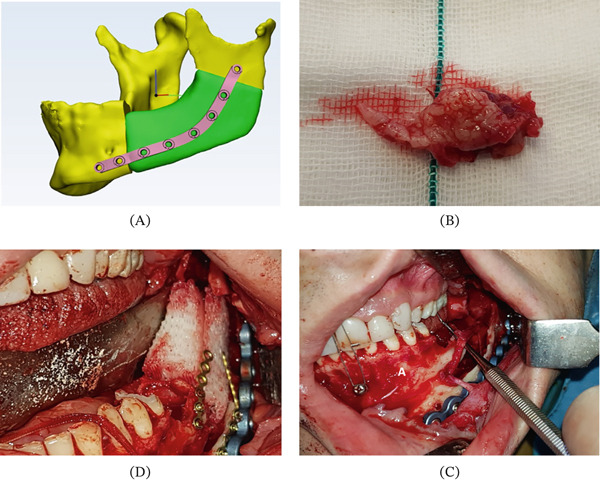
Workflow of treating Patient Number 2. (A) Design of the degradable PCL/TCP scaffold. (B) The resected 3 cm cemento‐ossifying fibroma. (C) Clinical view of the mandible after resection. Note the persistence of neurovascular bundle. (D) Implanting the scaffolds and fixing them with titanium miniplates and screws. Adapted from [23]. Used with permission from Springer Nature.

**Figure 4:**
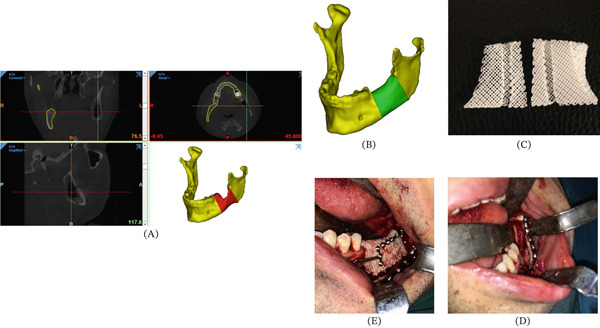
Workflow of treating Patient Number 3. (A) Preoperation radiographic data. (B) Design of the implant based on the contours of the contralateral side. (C) Fabricated PCL/TCP scaffold. Note the specified space for the neurovascular bundle. (D) Clinical view of the mandible after ablation procedure. Note the persistence of the inferior alveolar bundle. (E) Clinical view after fixing the scaffold with titanium miniplates.

**Figure 5:**
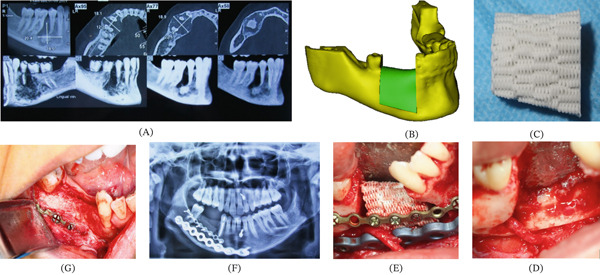
Workflow of treating Patient Number 4. (A) The presurgical radiography showed the presence of a 6 cm odontogenic keratocyst. (B) Design of the PCL/TCP scaffold. (C) Fabricated PCL/TCP scaffold. (D) Clinical view of the mandible after resection. Note the persistence of the neurovascular bundle. (E) Implant the scaffold and fix it with miniplates. (F) Postsurgical panoramic view (G) Postsurgical clinical view.

In Patients 1, 2, and 3, scaffolds were designed in two‐piece blocks to engulf the inferior alveolar neurovascular bundle within a predesigned cut‐in‐half passage (Figures 3, 4, and 5). For each patient, scaffold blocks were fitted into the surgical site and fixated on remnant bone tissue via miniplates and 2 mm titanium screws.

#### 2.3.3. Anatomic Bone Replacement

**Figure 6:**
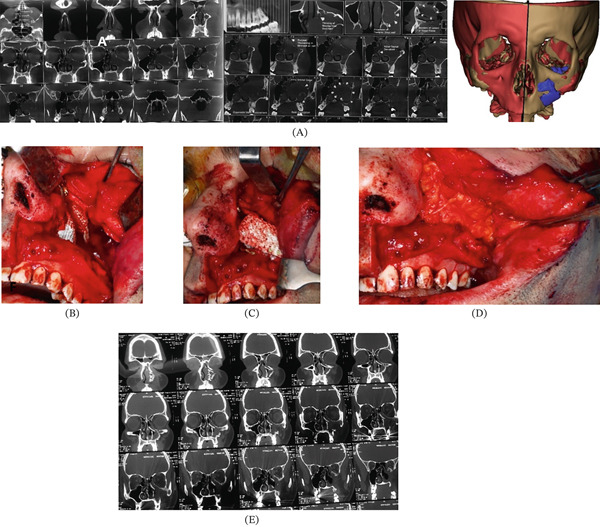
Workflow of treating Patient Number 5. (A) Preoperation radiographic data and designing the PCL/TCP scaffold, considering the contralateral contours. (B) Extracting the 7 cm odontogenic keratocyst and placing scaffolds in the defect. (C) Fixing the scaffold with titanium screws and miniplates. (D) Covering the scaffold with a pedicled buccal fat pad flap. (E) Eighteen months postoperation image.

**Figure 7:**
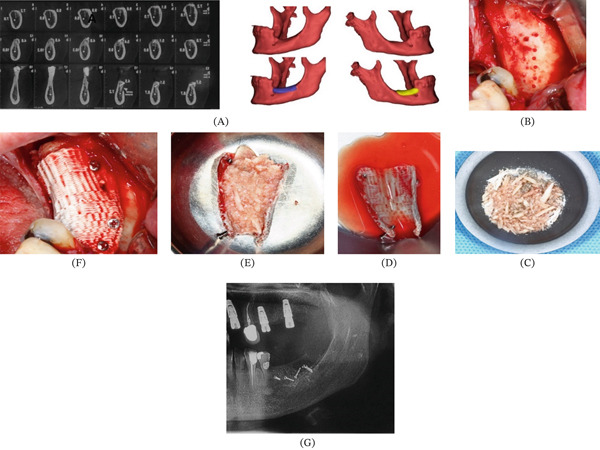
Workflow of treating Patient Number 6. (A) Preoperative radiographic data and design of the PCL/TCP scaffold to augment the atrophic posterior mandibular ridge. (B) Perforating the cortical bone in the recipient site. (C–E) Preparing the autogenous bone that was embedded in the scaffold. (F) Fixing the membrane using miniscrews. (G) Sixteen months postoperation radiography.

In Patient 4, the printed scaffold was implanted in the resected area following the same fixation protocol as above to separate the orbital cavity from the antrum. In Patient 5, the scaffold was filled with particulated autogenous bone and fixated on the mandibular alveolar ridge to preserve the underlying protected healing space, acting as a covering membrane (Figures 6 and 7).

### 2.4. Radiographic Analyses

New bone formation was calculated using pre‐ and postoperative CT images. Quantitative analyses were done using Mimics Software Version 20.0 (Materialise, Leuven, Belgium). To measure the amount of de novo bone formation, the pre‐ and postoperative 3D images were superimposed and registered based on anatomical landmarks. Next, the region of interest (ROI) was defined and dissected out. The volume of the separated section was analyzed in both models. The difference between pre‐ and postoperative defect volume was defined as the bone filling volume, and the bone formation ratio was defined using the following formula:

Bone formation %=ROI volume after follow−upROI volume before the surgery×100

Figure 2 shows the measurement protocol performed for a hypothetical sample. In Patients 4 and 5, who received anatomic scaffolds, the feasibility of space separation and formation of the bony plate was analyzed on CT images.

### 2.5. Histological Analyses

Histologic specimens were collected from functional bone replacement patients who had undergone dental implant surgery following detection of apt amounts of bone formation in radiographic analyses. Upon re‐entry, fixation screws were removed, and bone core biopsies were taken. Biopsies were fixed using 4% buffered formalin. Tris‐buffered EDTA was used for sample decalcification. Samples were passed through serial alcohol treatments and embedded in paraffin prior to cutting. A microtome (SCILAB Co. Ltd, Barnsley, United Kingdom) was used to cut specimen blocks into 5 *μ*m sections. Histological sections were stained with hematoxylin and eosin/Masson′s trichrome and inspected under a light microscope (E400, Nikon, 114 Japan).

## 3. Results

### 3.1. Clinical and Radiographic Outcomes

#### 3.1.1. Functional Bone Replacement–Bone Formation

In Patients 1, 2, and 3, PCL/TCP blocks were successfully implanted, and the neurovascular bundle was engulfed in its predesigned passage. The bone formation rate was within the range of 30%–75% (Figures 3, 4, and 5). The follow‐up period differed from 12 to 16 months (Table 1). Scaffold exposure occurred in one patient, possibly impeding bone formation. Patients 2 and 3 represented moderate levels of inflammatory reaction.

No complications related to mastication, speech intelligibility, or patient‐reported satisfaction were identified in any case. These observations were descriptive and based on routine clinical follow‐up records; standardized or validated functional outcome instruments were not used.

#### 3.1.2. Anatomical Bone Replacement

In Patients 4 and 5, there was no need for embedment of the neurovascular bundle. Appropriate space separation was seen in Patient 4 following hemilaminectomy after 18 months (Figure 6). Moreover, a thick and stable bony plate was inspected on the atrophic mandibular crest with a minimal concomitant inflammatory reaction in Patient 5 after 16 months (Figure 7). Considering the separating role of anatomic scaffolds, the bone formation rate and stability of the obtained bony plates were significant.

### 3.2. Histological Outcomes

**Figure 8:**
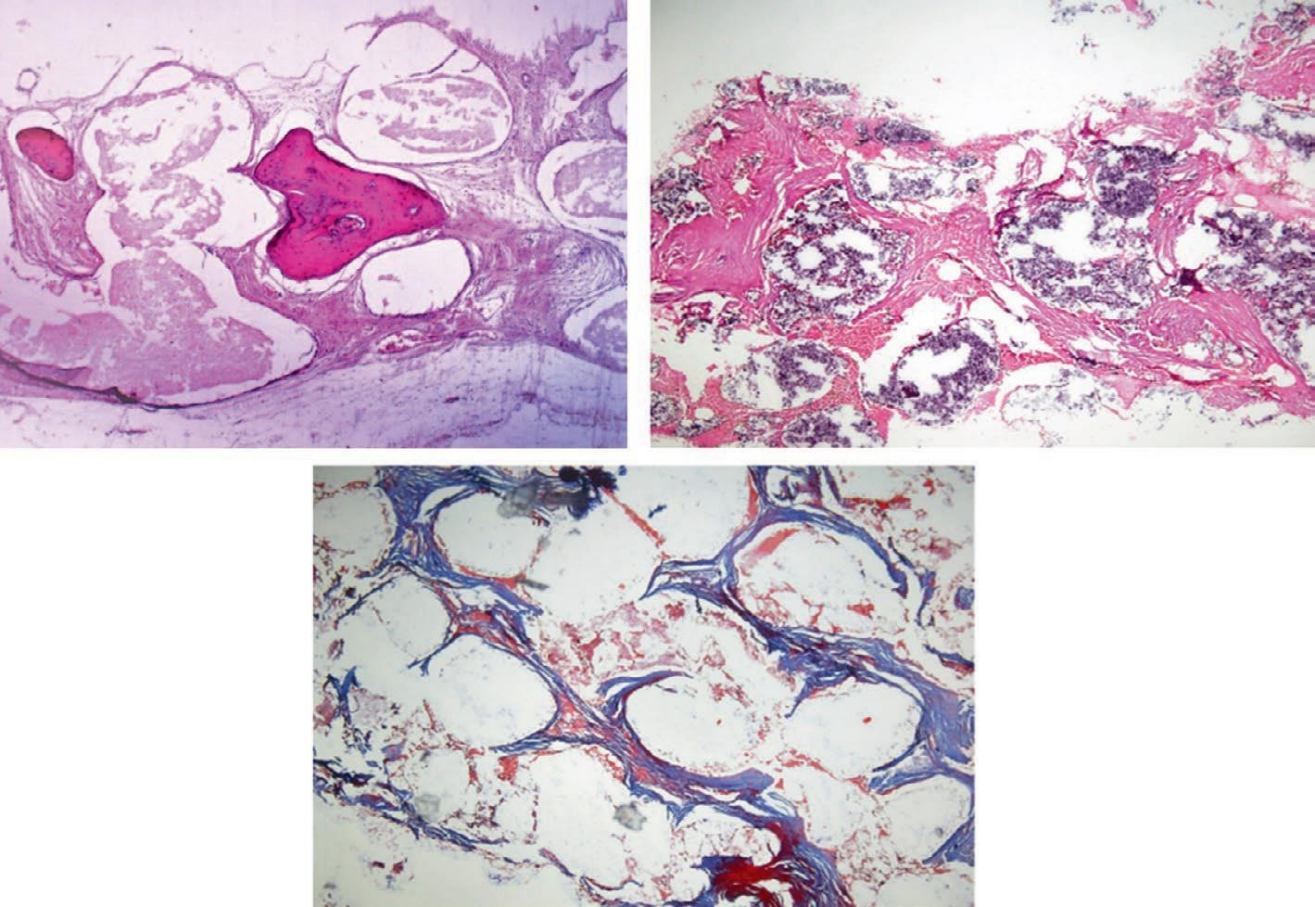
Histological sections of the biopsies obtained from patients who underwent implant insertion. Samples showed proper scaffold degradation and bone formation at x10 magnification.

Histological evaluations revealed the presence of osteocytes around Haversian canals. Signs of angiogenesis were also recognized, representing the successful formation of vital bone tissue. The presence of osteoclasts indicated active bone remodeling. The degrading biomaterial had properly integrated with de novo bone and the surrounding recipient tissue bed, indicating the scaffolds′ apt osteoconductive and osteoinductive abilities. Masson′s trichrome images showed penetration of collagen fibers into scaffold pores. Limited signs of inflammatory cell infiltration were observed in the samples (Figure 8).

## 4. Discussion

Functional bone replacement is an alternative option to treat maxillofacial bone defects that are more routinely treated using autografting techniques. In this study, we reported the application of this method in five patients with large maxillofacial bone defects. Patients who received functional scaffolds had different rates of new bone formation and scaffold degradation. Inherent defect specifics, such as size and morphology, may have contributed to the mentioned differences. Moreover, in patients who received anatomic scaffolds, proper space preservation and bone plate formation were seen.

Biodegradable scaffolds were previously implanted in the oral and maxillofacial region for regenerative purposes. However, most studies have employed scaffolds to reconstruct localized defects at minimally load‐bearing areas [2, 3, 5, 24–26]. To perform regeneration in large defects (i.e., > 6 cm), an intact soft tissue envelope and vascular sufficiency are vital. Patient‐specific bone scaffolds can have two general purposes: to restore and induce regeneration (functional bone replacement) or preserve the anatomic structures and spatially separate anatomic spaces (anatomic bone replacement).

Functional scaffolds were implanted in Patients 1, 2, and 3. Our results demonstrated a range of 30%–75% bone fill over 12–16 months of follow‐up in mandibular large defects. Jeong et al. [20] reported reconstruction of zygomaticomaxillary defects in eight patients using PCL/*β*TCP patient‐specific scaffolds. They observed 79.71% bone conformity after 12–24 months of follow‐up [20]. Their higher bone formation can be justified by superior blood supply and mechanical support against external forces in the maxilla relative to the mandibular regions. Maxillary biomechanics do not impose severe compressive and tensile forces on the zygomaticomaxillary region. However, the mandibular body often undergoes greater occlusal forces. Secured fixation can minimize the impact of imposed forces through load distribution. Defect location may challenge subsequent fixation in posterior regions [27].

Patient‐specific titanium mesh implants have been used as barriers for accurate reconstruction in the infraorbital and antral region after maxillary resection, depicting reliable long‐term results [28–30]. Although patient‐specific titanium meshes can prevent tissue entrapment and compression, the possibility of infection, delayed inflammation, hemorrhage, migration, and implant exposure encourages surgeons to consider alternative options such as osteoinductive degradable implants [31]. Application of these materials has been evaluated in animal studies, but there are limited reports concerning their clinical application [32, 33]. Kim [34] used PCL meshes for orbital wall repair in 22 patients. Although they reported proper repair in 21 cases, no quantitative data concerning the degradability or bony border formation was reported. In our study, two patients received anatomic scaffolds. One scaffold was placed to preserve the antrum space following hemimaxillectomy. Postoperative outcomes revealed feasible space separation after 18 months of follow‐up (Patient 4). Another patient received anatomic scaffolds as a membrane, resulting in guided in situ bone formation (Patient 5).

CAD/CAM technology enables surgeons to design the internal and external structure of the scaffold [35]. The external design accuracy contributes to dimensional accuracy and fitness, which can accelerate healing and diminish stress concentration [36]. An overall apt marginal adaptation between remnant defect walls and scaffolds was observed in our study. This obviated the need for intraoperative scaffold trimming and decreased operation time. In addition, CAD/CAM allows precise detection of the inferior alveolar neurovascular bundle to preserve it in a predesigned passage. Preserving the inferior alveolar neurovascular bundle in a benign odontogenic tumor of the mandible can provide blood supply and help the regeneration of segmental resected bone. Designing a passage was not discussed in the previous studies. Internal pore size and porosity of scaffolds can be adjusted via CAD/CAM to ensure tissue ingrowth and cell penetration, which our histological evaluations revealed accordingly.

A composite application of PCL and *β*TCP was sought to compensate for the PCL′s hydrophobicity and tailor the scaffolds′ degradation profile to match the natural rate of bone formation [16, 37–40]. Our results regarding the scaffold′s degradation profile were consistent with what is expected with pure PCL and pure TCP scaffolds, degrading over 2–4 years and 6–24 months, respectively. Our CT findings revealed that scaffolds had provided adequate osteoconductive properties and durability. Bone formation is expected to continue over future follow‐ups until the scaffold is fully degraded.

Scaffolds must not only reconstruct the deformities but also tolerate external forces within the physiologic range (i.e., physiologic occlusal force of 11–66 N [41]). The mechanical stability of PCL/*β*TCP scaffolds was evaluated in our previous in vitro analyses [19]. Scaffolds had mechanical stabilities enabling apt stability upon fixation and facile handling during implantation.

Dehiscence and implant exposure are serious and can lead to wound complications. Jeong et al. [20] had reported that only one out of eight patients was represented with implant exposure. The patient had undergone radiotherapy, and the incident was assumed to be associated with the patient′s compromised wound‐healing aptitude [20]. We had one case of scaffold exposure in a bone replacement model of the mandible and subsequent mild to moderate signs of infection and inflammation within the crestal area. However, it had limited adverse impacts on bone formation. Our main triggering factor was impaired oral hygiene.

PCL alone is reported to cause serious foreign body reactions, leading to long‐term late‐onset inflammatory complications (i.e., formation of granulomatous lesions, late allergic responses, and chronic inflammation consequent to PCL‐based dermatologic fillers) [42–45]. To overcome this issue, mineral material is added to the blend to neutralize the acidic environment. Our patients represented a detectable, yet not severe, clinical inflammatory phenomenon, which was managed uneventfully. The severity of immunologic reactions is also associated with patients′ inflammatory status.

## 5. Limitations

Reported results regarding bone formation and degradation rates are not final and expected to continue over prolonged follow‐ups. Therefore, short‐term follow‐up bars make final conclusions. In addition, the gold standard method for specifying degradation and bone formation rates is histological evaluation. However, only three patients received implants over the course of this study. Moreover, the selection of proper biomaterials and techniques for clinical functional bone regeneration necessitates further studies with larger sample sizes.

Although the same CT‐based workflow was applied consistently across all cases, volumetric assessment based on image registration, ROI definition, and segmentation remains partly operator‐dependent and may be influenced by segmentation variability. Therefore, the radiographic measurements should be interpreted as approximate volumetric estimates rather than absolute values.

In addition, functional outcomes, including mastication, speech, and patient satisfaction, were documented descriptively from clinical records and were not assessed using standardized or validated instruments.

## 6. Conclusion

PCL/*β*TCP scaffolds showed proper biological and physicochemical behaviors upon implantation in the maxillomandibular region, making them a potential supplement and/or alternative to autogenous bone grafting in selected patients in the future. However, possible complications, such as inflammation or graft and scaffold exposure, must be noted. Further studies are warranted considering histologic outcomes, long‐term complications, and the selection of proper biomaterials and techniques.

## Author Contributions

**Hekmat Farajpour:** data curation, formal analysis, data interpretation. **Helia Sadat Haeri Boroojeni:** data curation, writing – original draft. **Sadra Mohaghegh:** data curation, writing – original draft. **Hanieh Nokhbatolfoghahaei:** data curation, formal analysis, data interpretation. **Arash Khojasteh:** conceptualization, study design, writing – review and editing, critical revision of the manuscript.

## Funding

No funding was received for this manuscript.

## Disclosure

All provided materials are original, and no permission is required from other sources.

## Ethics Statement

The study was performed according to the World Medical Association Declaration of Helsinki′s declared ethical principle under the approval of the Ethical Committee of the Shahid Beheshti University of Medical Sciences (IR.SBMU.RETECH.REC.1398.377).

## Consent

All patients were informed about surgical procedures, treatment options, and potential complications prior to signing informed consent.

## Conflicts of Interest

The authors declare no conflicts of interest.

## Data Availability

The data that support the findings of this study are available upon request from the corresponding author. The data are not publicly available due to privacy or ethical restrictions.
